# Echotextural characteristics of the mammary gland during early lactation in two breeds of sheep varying in milk yields

**DOI:** 10.21451/1984-3143-AR2019-0025

**Published:** 2019-11-18

**Authors:** Maciej Murawski, Tomasz Schwarz, Mark Jamieson, Pawel Mieczyslaw Bartlewski

**Affiliations:** 1 Agricultural University of Cracow Department of Animal Biotechnology Kraków Poland Agricultural University of Cracow, Department of Animal Biotechnology, Kraków, Poland; 2 Agricultural University of Cracow Department of Swine and Small Animal Breeding Kraków Poland Agricultural University of Cracow, Department of Swine and Small Animal Breeding, Kraków, Poland; 3 University of Guelph Ontario Veterinary College Department of Biomedical Sciences Guelph Canada University of Guelph, Ontario Veterinary College, Department of Biomedical Sciences, Guelph, Canada

**Keywords:** sheep, milk, lactation, mammary gland, ultrasonography

## Abstract

The main goal of this preliminary study was to determine and compare ultrasonographic characteristics of the mammary gland in two genotypes of ewes varying in milk productivity at 2, 3 and 4 weeks after lambing. Ultrasonographic images of the udder were obtained using the 5.0- and 7.5-MHz transducers, in axial and coronal planes, in four low milk-yielding Polish Mountain sheep and six high milk-yielding Olkuska ewes. All ultrasonograms were subjected to computerized image analyses using commercially available image analytical software (Image ProPlus ^®^ ; Media Cybernetics Inc., San Diego, CA, USA) to determine numerical pixel values (NPVs) and heterogeneity (pixel standard deviation-PSD) of the mammary gland parenchyma. During the 28-day period post-partum, the Olkuska sheep exceeded (P < 0.05) Polish Mountain ewes in milk productivity (31.6 ± 2.7 l and 25.0 ± 4.2 l, respectively; means ± SEMs) as estimated by the mean weight gains of suckling lambs. In animals examined with the 5.0-MHz transducer, mean NPVs of the mammary gland parenchyma in Olkuska ewes and mean PSD in both genotypes of ewes were lower (P < 0.05) before than after milking. In addition, PSD recorded both before and after milking were lower (P < 0.05) in the Polish Mountain compared with Olkuska breed. Mean PSD values for the mammary gland were less (P < 0.05) before than after milking in Polish Mountain ewes and they were greater (P < 0.05) in Olkuska compared with Polish Mountain ewes examined with the 7.5-MHz probe after milking. It can be concluded that milk quantity, histomorphology of the udder and ultrasound transducer frequency may all impinge on the echotextural characteristics of the mammary parenchyma in different breeds of sheep. Our observations warrant future studies of correlations between milk composition, mammary gland histophysiology and ultrasonographic image attributes of the mammary gland in ruminants.

## Introduction

Grey-scale ultrasonographic images are composed of numerous brightness elements called pixels corresponding to multiple acoustic interfaces within the examined tissue (the boundaries between regions of different physicochemical properties; Ahmadi et al., 2013 ). Ultrasound transducers contain piezoelectric crystals emitting high-frequency sound waves that are modified as they encounter the acoustic interfaces. Ultrasound beams can be attenuated as they traverse tissues, or they can be scattered or deflected by the scanned objects and their integral components. Scattered or reflected waves received by the transducer determine the relative intensity of each pixel ( Wu et al., 2009 ). Numerical pixel values (NPVs), a quantitative measure of pixel brightness, can be determined objectively using several image analytical software packages. Computerized analysis of ultrasonograms increases precision of measurements and the range of detectable intensity variations onto those that cannot be detected with the naked eye ( Giffin et al., 2009 ). The NPV is a unitless parameter that ranges from 0 (absolute black) to 255 (absolute white) and pixel heterogeneity is defined as the standard deviation of NPVs ( Giffin et al., 2009 ). Both echotextural variables are objective measures of tissue echogenic properties and valuable indicators of corresponding histophysiological changes ( Giffin et al., 2009 ; Ahmadi et al., 2013 ).

Ultrasound images of the mammary gland in ruminant species have been described as exhibiting granular texture due to the presence of less echoic lobular alveoli being dispersed within hyperechoic connective tissue ( Olechnowicz and Jaśkowski, 2009 ; Santos et al., 2015 ; Barbagianni et al., 2017 ). The mammary parenchyma in healthy dairy goats appears as a hyperechoic structure with slightly coarse echotexture ( Fasulkov, 2012 ; Santos et al., 2015 ; Barbagianni et al., 2017 ). Occasionally, anechoic lactiferous ducts or larger blood vessels are identified during the scanning of the udder ( Ruberte et al., 1994 ; Nudda et al., 2000 ). No studies to the best of our knowledge have quantitatively described the mammary gland parenchyma using precise measurements of echotextural variables in lactating ewes varying in milk yields.

Therefore, the goals of this preliminary ultrasonographic study were to describe quantitative echotextural changes in the mammary gland parenchyma of low and high milk-yielding ewes at three different time points during the post-parturient period (at weekly intervals from 2 weeks after lambing until 4 weeks post-partum) and to examine additional factors that can impinge on mammary gland echotexture (e.g., scanning plane and the type of an ultrasound transducer used). Most ewes peak in their milk production around three to four weeks after the lambs are born ( Molik et al., 2008 ). We hypothesized that quantitative ultrasonographic characteristics of the mammary gland in the ewes of both genotypes would mainly reflect the varying rates of production and accumulation of its excretory product. The results of this study can also serve as a set of reference values to standardize computerized image analysis of the normal ovine mammary gland ultrasonograms.

## Methods

### Animals and locality

All experimental procedures followed the EU Directive 2010/63/EU for animal experimentation and the Polish law for the care and use of animals in research (2 August 1997). This experiment was conducted in the Experimental Station of the Department of Animal Biotechnology of the Agricultural University of Cracow, Poland situated in Bielany (latitude: 52°17'32.71” N, longitude: 20°56'7.12” E) and it utilized a total of ten clinically healthy, multiparous ewes (four Polish Mountain sheep and six Olkuska ewes) aged 2.5 to 4.5 years and weighing 60 ± 5 kg. The Polish Mountain sheep is a wool-yielding and dairy breed of sheep maintained mainly in southern Poland (Podhale region; Gebarowska et al., 1996 ). The Olkuska breed is a product of cross breeding of Polish Pomeranian ewes, Friesian and Kent rams, and domestic breeds raised in the Poland’s Małopolska (Lesser Poland) region. The Olkuska sheep are used mainly for milk production, and their lambs are fast-growing and early-maturing compared with other genotypes of long-wool sheep ( Gebarowska et al., 1996 ; Zięba et al., 2002 ; Bartlewski et al., 2017 ). During the lactation, from Days 2 to 28 after lambing, the prolific Olkuska ewes exceed Polish Mountain sheep in net milk production by approximately 18 l/ewe (52.1 l vs. 34.2 l, respectively; Molik et al, 2008 ). The ewes and lambs were housed in a barn under natural conditions of photoperiod and ambient temperature. Throughout the entire experimental period, the animals received a diet formulated to provide 100% of metabolic requirements during gestation and lactation, in compliance with the guidelines of the Polish National Research Institute for Animal Production ( Norms, 1993 ). Water, hay and mineral salt licks were available *ad libitum* .

### Estrous synchronization, breeding and lambing

Estrus was synchronized with intravaginal sponges containing 45 mg of flugestone acetate (Chronogest ^®^ ; MSD Animal Health, Boxmeer, Holland) inserted for 14 days in the month of August (transition from the anestrous period to the breeding season). On the day of sponge withdrawal, all ewes received a single i.m. injection of 400 IU of equine chorionic gonadotropin (eCG; Folligon ^®^ , Intervet Int., Warsaw, Poland) and were subsequently bred by two Olkuska rams. The spontaneous (non-induced) lambing occurred between 20-25 January of the following year.

### Lactation, ultrasonographic examinations of the udder and computerized image analysis

**Figure 1:**
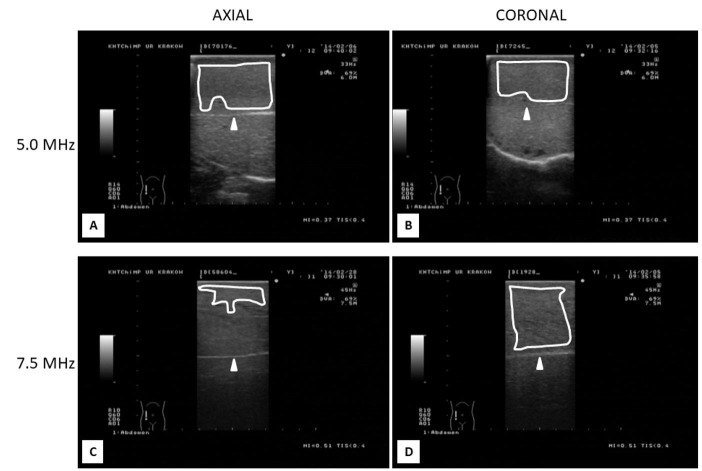
Photographic reproductions of mammary gland ultrasonograms from lactating ewes with the udder scanned in an axial (A and C) and coronal (B and D) plane using either 5.0- (A and B) or 7.5-MHz (C and D) linear-array transducers. White arrowheads indicate the location of the connective tissue septum separating the two halves of the udder. Delineated areas (white lines) represent the potential placement of computer-generated polygonal meters used for echotextural analyses of the mammary parenchyma (Image ProPlus ^®^ analytical software).

From Days 2 to 28 post-partum (i.e., when suckling was the only source of nutrition for lambs), the total milk yield was estimated using a formula: 4.5 l of milk equals to 1 kg of lamb body weight gain ( Molik et al., 2008 ). Sheep udders were examined ultrasonographically immediately before and after complete hand milking. Both halves of the udder (left and right) were scanned using two different types of linear-array transducers (5.0- and 7.5-MHz) connected to the Aloka ProSound 2 scanner (Hitachi Aloka Medical Ltd., Tokyo, Japan), in the axial and coronal planes of the middle portion of the mammary gland ( Figure 1 ). All examinations were performed at constant settings of the ultrasound scanner for main gain, contrast, focal points and TGC (Time-Gain Compensation) parameters. Animals were restrained in a standing position and an aqueous gel was applied as a coupling material between the surface of the transducer and the skin of the udder. All ultrasonograms were initially stored as DICOM (Digital Imaging and Communications in Medicine) files but the 2D still images of the mammary gland (*.TIFF) were subsequently saved at standardized settings, with a resolution of 720 x 480 pixels and 256 shades of grey.

Computerized image analysis of the mammary gland parenchyma was conducted using commercially available image analytical software (Image ProPlus ^®^ ; Media Cybernetics Inc., San Diego, CA, USA) in the Department of Biomedical Sciences, University of Guelph, ON, Canada. One polygon meter placed above the connective tissue septum separating the left and right halves of the udder was used to determine echotextural variables of the mammary gland parenchyma ( Figure 1 AD); a special care was taken to omit apparent reflection artifacts, large blood vessels and lactiferous ducts. The mean numerical pixel values (NPVs) and pixel heterogeneity (standard deviation of numerical pixel values-PSD) for each region of interest were computed.

### Data analysis and statistical comparisons

Statistical tests were performed using the SigmaPlot™ computer program (version 11.0; Systat Software Inc., Richmond, CA, USA). Single time-point observations were compared between the two breeds of sheep by Student *t* -test. Changes in echotextural variables were analyzed by multivariate analysis of variance (ANOVA). The main effects included into the statistical model were breed, time post-partum, scanning order (i.e., before or after milking), side (left vs. right half) and scanning plane (axial vs. coronal). The data for ultrasonographic images obtained with the 5.0-MHz or 7.5-MHz transducers were analyzed separately. If the main effects or their interactions were significant, further analyses of differences between individual mean values were done using the least significant difference (LSD) test. A P value < 0.05 was considered statistically significant. All results are presented as mean ± standard error of the mean (SEM).

## Results

### Lambing and lactation

The number and birth weights of lambs as well as their weights recorded 28 days after lambing and estimated milk yields of dams are given in Table 1 . In spite of similar lamb productivity, the Olkuska breed lambs exhibited greater (P < 0.05) weight gain and hence the Olkuska ewes had greater estimated milk productivity compared with the Polish Mountain sheep during the four-week period post-partum.

**Table 1:** Lamb characteristics and estimated milk yields during the first 28 days of lactation in Polish Mountain and Olkuska ewes.

Breed	Number of lambs	Birth weight (kg)	Body mass (Day 28 post-partum; kg)	Estimated milk yield (l) *
Polish Mountain sheep (n=4)	1	4	9.4	24.3
	1	3.5	6.5	13.5
	2	4.6/3.8	8.6/6.4	29.7
	2	4.8/4.8	8/8.8	32.4
Mean±SEM	1.5 ± 0.3	4.2 ± 0.2	7.9 ± 0.5 ^a^	25.0 ± 4.2 ^a^
Olkuska sheep (n=6)	2	3.5/3.1	7.5/7.0	35.5
	1	5.5	12.6	31.9
	1	4.0	11.2	32.4
	1	3.5	11.0	33.7
	1	3.8	8.0	18.9
	2	3.5/3.2	7.6/7.4	37.3
Mean±SEM	1.3 ± 0.2	3.8 ± 0.3	9.0 ± 0.3 ^b^	31.6 ± 2.7 ^b^

* Milk productivity calculated using the equation: 1 kg of lamb weight gain = 4.5 l of milk;

ab P < 0.05 (within columns means denoted by different letter superscripts vary significantly).

### Factors that impinged on echotextural characteristics of the mammary gland

There was no significant effect of scanning plane (i.e., axial vs. coronal) or left vs. right udder half on the echotextural attributes of the mammary gland in the ewes of the present study. Therefore, all echotextural data were analyzed on a per animal basis (i.e., numerical pixel intensity values (NPVs) and pixel heterogeneity (PSD) obtained for the two halves of the udder in two different planes were averaged to give an overall mean of the respective mammary gland).

**Figure 2:**
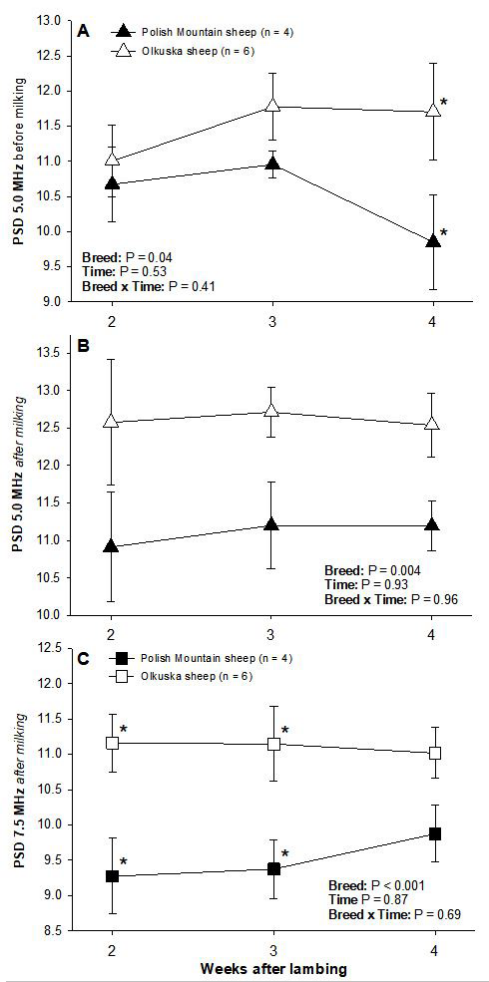
Significant differences in echotextural characteristics of sheep mammary glands (NPVs-numerical pixel values a.k.a. pixel intensity or brightness, and PSD-standard deviation of NPVs or pixel heterogeneity). Data were collected weekly with the use of the 5.0-MHz or 7.5-MHz linear array transducer before and after milking of Polish Mountain (n = 4) and Olkuska ewes (n = 6) at 2, 3 and 4 weeks post-partum). Asterisks indicate significant differences between the two breeds.

Using the 5.0-MHz transducer, there were significant main effects of the scanning order (i.e., before and after milking) for NPVs and PSD values of the mammary gland parenchyma. Mean NPVs in Olkuska ewes and mean PSD values in both genotypes of ewes were lower (P < 0.05) before than after hand milking ( Table 2 ). In addition, PSD recorded both before and after milking were lower (P < 0.05) in the Polish Mountain compared with Olkuska sheep ( Table 2 , Figure 2 ); the difference in PSD values before milking was most prominent (P < 0.05) on Week 4 post-partum ( Figure 2 A).

**Table 2:** Summary of differences in echotextural properties of the mammary gland parenchyma in Polish Mountain and Olkuska ewes examined ultrasonographically at 2,3 and 4 weeks after lambing.

Scanning order/Breed	Polish Mountain sheep (n=4)		Olkuska sheep (n=6)	
	NPVs	PSD	NPVs	PSD
5.0-MHz transducer				
Before milking	81.2 ± 2.8	10.5 ± 0.3 ^Aa^	79.1 ± 1.2 ^a^	11.5 ± 0.2 ^Ba^
After milking	82.3 ± 1.3	11.1 ± 0.1 ^Ab^	83.7 ± 0.9 ^b^	12.6 ± 0.1 ^Bb^
7.5-MHz transducer				
Before milking	69.0 ± 0.4	10.1 ± 0.3 ^a^	71.5 ± 1.0	9.9 ± 0.4
After milking	72.6 ± 1.9	11.1 ± 0.05 ^Ab^	72.8 ± 1.4	9.5 ± 0.2 ^B^

NPVs-numerical pixel values; PSD-pixel standard deviation or heterogeneity. Different letter superscripts indicate statistical differences (P < 0.05) between mean values: ^AB^ between breeds and ^ab^ before and after milking.

In animals examined with the 7.5-MHz probe, mean PSD values of the mammary gland parenchyma were less (P < 0.05) before than after milking in Polish Mountain ewes ( Table 2 ). In addition, they were greater (P < 0.05) in Olkuska compared with Polish Mountain ewes at 2 and 3 weeks post-partum ( Table 2 , Figure 2 C). There was no effect of Time (i.e., weeks post-partum) on any of the ultrasonographic characteristics of the mammary gland in this study.

## Discussion

Mammary gland NPVs obtained with the 5.0-MHz transducer were significantly greater after than before milking in Olkuska sheep and pixel heterogeneity (PSD) values were significantly greater after than before milking in both genotypes of ewes examined with the 5.0-MHz probe and in Polish Mountain ewes scanned with the 7.5-MHz transducer. These differences in relative sensitivity of the two transducers are difficult to explain. A recent study, however, has shown that the low-frequency transducer (frequency range of 1 to 5 MHz) is more sensitive than the high-frequency transducer (5 to 12 MHz) in detecting malignant histopathological changes using breast contrast-enhanced ultrasound (CEUS; Wang et al., 2016 ); the authors suggested that detection effectiveness of the diagnostic transducers used might be influenced by the physical properties (i.e., resonance frequency) of a contrast agent. Future studies are needed to clarify specific associations among ultrasound scanning frequencies, recordable tissue/liquid excretory product echotexture and detection efficacy of ultrasonographic imaging.

An increase in the mean pixel intensity of the udder after complete milking of Olkuska sheep was most likely due to the removal of the hypoechoic excretory product ( Barbagianni et al., 2015 ). However, we expected PSD values to decrease after removal of residual milk, which *in situ* potentially generates greater heterogeneity due to the presence of more echoic “permanent” parenchymal tissues such as collagen and mammary epithelial cells (MECs; Suárez-Trujillo et al., 2013 ; Lérias et al., 2014 ; Barbagianni et al., 2015 ).

Mean pixel heterogeneity (PSD) recorded both before and after milking were generally lower in Polish Mountain compared with Olkuska sheep (with an exception of PSD before milking in ewes monitored with the 7.5-MHz transducer). The difference in mammary gland PSD values between the two breeds before milking may be, at least partly, due to the relative volume of milk stored in the mammary gland. Dairy ewes can store 50 to 75% of their milk in the gland cisterns (meaning that 25 to 50% of milk remains in secretory lobules/alveoli and small excretory ducts (interlobular and lobular) within the mammary gland parenchyma; Makovický et al., 2013 ). In dairy cows, however, the difference in mammary gland echotexture before and after milking was only minimal ( Szencziová and Strapák, 2012 ); those results are surprising as approximately 60 to 80% of milk in dairy cows is stored in the mammary parenchyma. Additionally, lambs were kept with their mothers and continuous suckling was permitted in the present study. Therefore, this variation in parenchymal echotexture may be also due to the differences in parenchymal tissue microstructure and/or milk chemical composition between the two breeds of sheep studied.

It is, however, unclear what specific features of the udder morphology could affect echotextural properties recorded after milking in lactating ewes. It can be speculated that echogenic cells or tissues that undergo the most profound changes during lactopoiesis and lactogenesis are primarily responsible for the shifts in mammary gland echotexture ( Suárez-Trujillo et al., 2013 ; Lérias et al., 2014 ). MECs are required for lactogenesis and most mammary epithelial cell hyperplasia in sheep occurs during late puberty and late pregnancy, while little occurs during lactation ( Paten, 2014 ). Barbagianni et al. (2015) measured grey scale intensity of the mammary gland in Chios-cross ewes from 120 days of pregnancy to early lactation and observed a significant increase in pixel intensity up to parturition, followed by a significant decline in numerical pixel values after the onset of milk production. An increase in pixel intensity prior to parturition can be associated with MEC hyperplasia in late pregnancy because cell nuclei are the most dominant subcellular source of ultrasound wave scattering ( Giffin et al., 2014 ). The Olkuska and Polish Mountain sheep differ in milk yields. Akers et al. (2006) compared udder morphology of beef and dairy cows over pregnancy and lactation and observed that MECs were more abundant and significantly more differentiated in dairy compared with beef cows. Hence, the inherent difference in MEC populations may partially explain echotextural differences observed in the ewes of the present study.

Protein and lipid content of the tissue may determine its ultrasonographic appearance ( Ahmadi et al., 2013 ). Significant correlations exist between ultrasonographic image characteristics and chemical composition of the testes ( Ahmadi et al., 2013 ) and skeletal muscles ( Clague et al., 1995 ; Schwarz et al., 2019 ). Mean PSD values of testicular tissue in the ram were positively correlated with extractable lipids ( Ahmadi et al., 2013 ). In the milk samples from the first 30-day sampling period post-partum, fat concentrations were significantly greater in Olkuska ewes compared with Polish Mountain sheep ( Molik et al., 2008 ). Therefore, quantitative changes in milk composition may also account for the difference in mammary gland echotexture between the two breeds of sheep in this study.

In summary, ultrasonographic image attributes of sheep mammary parenchyma determined with computer-assisted image analyses were affected by the breed/milk yield of lactating ewes, ultrasound transducer used (5.0- vs. 7.5-MHz), and scanning order (before vs. after complete milking), but not by the time elapsed from lambing (2 to 4 weeks post-partum). Variations in milk composition and intrinsic histophysiological characteristics of the mammary gland may both affect its echotexture in lactating ewes. Our present observations warrant future studies of the correlations between milk composition and ultrasonographic image attributes of the mammary gland in ruminant species.
